# Performance of the deep convolutional neural network based magnetic resonance image scoring algorithm for differentiating between tuberculous and pyogenic spondylitis

**DOI:** 10.1038/s41598-018-31486-3

**Published:** 2018-09-03

**Authors:** Kiwook Kim, Sungwon Kim, Young Han Lee, Seung Hyun Lee, Hye Sun Lee, Sungjun Kim

**Affiliations:** 1 Department of Radiology, Gangnam Severance Hospital, Yonsei University College of Medicine, Research Institute of Radiological Science, Center for Clinical Imaging Data Science, Seoul, South Korea; 2 Department of Radiology, Severance Hospital, Yonsei University College of Medicine, Research Institute of Radiological Science, Center for Clinical Imaging Data Science, Seoul, South Korea; 3 Department of Radiology, National Health Insurance Service Ilsan Hospital, Goyang-si, Gyeonggi-do, South Korea; 4 Biostatistics Collaboration Unit, Research Center for Future Medicine, Yonsei University College of Medicine, Seoul, South Korea

## Abstract

The purpose of this study was to evaluate the performance of the deep convolutional neural network (DCNN) in differentiating between tuberculous and pyogenic spondylitis on magnetic resonance (MR) imaging, compared to the performance of three skilled radiologists. This clinical retrospective study used spine MR images of 80 patients with tuberculous spondylitis and 81 patients with pyogenic spondylitis that was bacteriologically and/or histologically confirmed from January 2007 to December 2016. Supervised training and validation of the DCNN classifier was performed with four-fold cross validation on a patient-level independent split. The object detection and classification model was implemented as a DCNN and was designed to calculate the deep-learning scores of individual patients to reach a conclusion. Three musculoskeletal radiologists blindly interpreted the images. The diagnostic performances of the DCNN classifier and of the three radiologists were expressed as receiver operating characteristic (ROC) curves, and the areas under the ROC curves (AUCs) were compared using a bootstrap resampling procedure. When comparing the AUC value of the DCNN classifier (0.802) with the pooled AUC value of the three readers (0.729), there was no significant difference (*P* = 0.079). In differentiating between tuberculous and pyogenic spondylitis using MR images, the performance of the DCNN classifier was comparable to that of three skilled radiologists.

## Introduction

Diagnoses of infectious spondylitis have risen in number due to various causes: increasing numbers of adults of older age or immunocompromised patients, universal use of invasive spinal procedures or surgeries, and improving accuracy of imaging diagnosis, owing to the revolutionary development of imaging diagnostic methods^1^. Because rapid anti-tuberculosis treatment can prevent future disability^2^, it is important to differentiate between tuberculous and pyogenic spondylitis, which are the most common causes of infectious spondylitis. As magnetic resonance (MR) imaging is the most effective method for diagnosing infectious diseases of the spine, because of its superior soft tissue contrast, there have been various studies on differentiating between tuberculous and pyogenic spondylitis using MR imaging^3–6^.

Early stage disease and atypical image phenotypes make it difficult to establish a robust MR imaging feature that can distinguish tuberculous spondylitis from pyogenic spondylitis. Distinguishing between the two using MR images alone remains a challenging task, even for skilled radiologists^7^. To the best of our knowledge, few investigations have sought to demonstrate the diagnostic performance of MR imaging on differentiating between tuberculous and pyogenic spondylitis. Although MR imaging alone could be insufficient for differential diagnosis^8,9^, MR imaging is still one of the most important diagnostic tests performed before applying invasive procedures.

Recently, the deep convolutional neural network (DCNN) has been shown to have high performance in the field of computer vision^10–12^, and its application in the medical field is being actively studied, especially in radiology fields^13–16^. DCNN extracts low- to high-level features from the training images and uses them to select the most important features for solving a given task^17^. Considering the ability of DCNN to distinguish complex objects in the ImageNet challenge, we postulated that DCNN could show good performance in discriminating between tuberculous and pyogenic spondylitis.

The purpose of this study was to evaluate the performance of the DCNN in differentiating between tuberculous and pyogenic spondylitis on MR images, compared with the performance of three skilled radiologists.

## Results

The baseline demographic and clinical characteristics of the patients are listed in Table 1. The median ages of the patients with tuberculous or pyogenic spondylitis were 59 (Interquartile Range, 38–71) and 64 (Interquartile Range, 56–72) years, respectively. The frequency of fevers was significantly higher among patients with pyogenic (43.2%) than tuberculous spondylitis (12.5%) (*P* < 0.001). More specifically, the frequency of intermittent fevers was significantly higher in febrile patients with tuberculous than pyogenic spondylitis (*P* < 0.048). In patients with back pain, the incidence of that lasting more than 4 weeks was significantly higher in patients with tuberculous than pyogenic spondylitis (*P* < 0.001). Patients with pyogenic spondylitis showed significantly higher serum erythrocyte sedimentation rate, C-reactive protein, and procalcitonin levels than those with tuberculous spondylitis.

**Table 1:** Comparison of baseline demographics and clinical characteristics between patients with tuberculous or pyogenic spondylitis.

Variables^†^	Tuberculous (n = 80)	Pyogenic (n = 81)	P Value^‡^
Female, n (%)	49 (61.3)	40 (49.4)	0.130
Median age (y), median (IQR)	59 (38–71)	64 (56–72)	0.011
**Clinical features, n (%)**			
Fever	10 (12.5)	35 (43.2)	<0.001
Intermittent fever	5	5	0.048^*^
Back pain	69 (86.3)	75 (92.6)	0.108
Acute (≤4 weeks)	27	61	<0.001^*^
Subacute or chronic (>4 weeks)	42	15	<0.001^*^
Neurological symptom	29 (36.3)	40 (49.4)	0.092
**Baseline laboratory results, median (IQR)**			
Hematocrit	36.9 (34.6–39.5)	34.7 (31.8–37.5)	0.001
WBC	7,285 (5,835–9,295)	8,720 (6,890–11,970)	<0.001
% Neutrophils	70.4 (62.4–75.4)	75.8 (68.4–84.5)	0.001
ESR	61.5 (42.0–94.8)	79 (57–105)	0.014
CRP	23.8 (7.8–48.7)	56.3 (19–179.9)	<0.001
Procalcitonin	0.08 (0.06–0.24), 16/80	0.17 (0.07–0.79), 40/81	0.040
Albumin	3.9 (3.6–4.2)	3.4 (2.9–3.8)	<0.001

Abbreviations: IQR, interquartile range; WBC, white blood cell; ESR, erythrocyte sedimentation rate; CRP, C-reactive protein.

^†^Values represent the number of subjects (%) or median (IQR).

^‡^Values were obtained using Student t, Fisher exact, or chi-square test as appropriate.

*p values for subgroup comparison.

The diagnostic performance of the DCNN classifier based on the four-fold cross validation was as follows. The mean deep-learning (DL) score of tuberculous spondylitis patients was greater than that of pyogenic spondylitis patients (0.570 [0.513–0.628] versus 0.266 [0.214–0.317], p < 0.001) using the DCNN classifier. The overall area under receiver operating characteristic curves (AUC) of the DCNN classifier, considering all subgroups together, was 0.802 (95% confidence interval, 0.733–0.872). The AUC values for each subgroup used for each-fold cross validation are summarized in Table 2.

**Table 2:** Area under the receiver operating characteristics curve for the deep convolutional neural network classifier based on four-fold cross validation.

	**AUC**	**95% CI**	**Sensitivity/Specificity (%)** ^**†**^
Subgroup 1	0.723	(0.563–0.883)	
Subgroup 2	0.856	(0.723–0.989)	
Subgroup 3	0.853	(0.732–0.973)	
Subgroup 4	0.761	(0.606–0.916)	
**Total**	0.802	(0.733–0.872)	85.0/67.9

Abbreviations: AUC, area under the receiver operating characteristics curve; CI, confidence interval.

^†^Optimal threshold by the Youden index.

At the optimal cutoff point, the sensitivity, specificity, and accuracy of the DCNN classifier for all participants were 85.0, 67.9, and 76.4% (threshold of DL score = 0.313, Youden index). Those of the three radiologists were 72.5, 67.9, and 70.2; 72.5, 66.7, and 69.6; and 70.0, 71.6, and 70.8% (threshold of 5-point confidence scale score = 3.5, Youden index), respectively. Of 161 patients, the DCNN classifier correctly classified 123 patients, and the three radiologists correctly classified 113, 112, and 114 patients (Table 3).

**Table 3:** Comparison of the diagnostic performances of the deep convolutional neural network classifier and three radiologists derived from the confusion matrix.

	Sensitivity (%) (95% CI)	TP/TP + FN	Specificity (%) (95% CI)	TN/TN + FP	Accuracy (%) (95% CI)	TP + TN/TP + FN + TN + FP
DCNN	85.0 (74.9~91.7)	68/80	67.9 (56.5~77.6)	55/81	76.4 (69.1~82.7)	123/161
Reader 1	72.5 (61.9~81.1)	58/80	67.9 (56.5~77.6)	55/81	70.2 (63.1~77.3)	113/161
Reader 2	72.5 (61.9~81.1)	58/80	66.7 (55.9~76.0)	54/81	69.6 (62.5~76.7)	112/161
Reader 3	70.0 (59.2~78.9)	56/80	71.6 (61.0~80.3)	58/81	70.8 (63.8~77.8)	114/161

Abbreviations: CI, confidence interval; TP, true positive; FN, false negative; TN, true negative; FP, false positive; DCNN, Deep convolutional neural network classifier.

**Figure 1:**
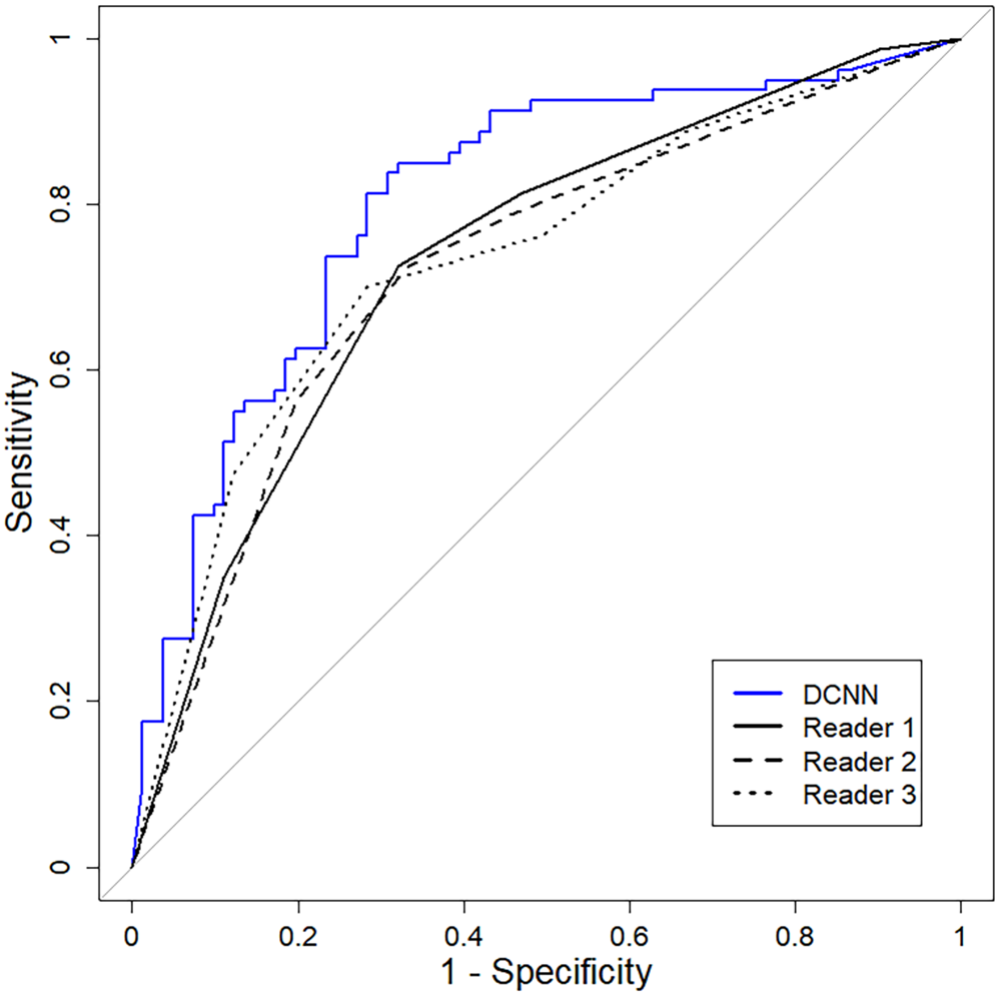
Receiver operating characteristic curves of the deep convolutional neural network (DCNN) classifier and three radiologists.

The AUC value of the DCNN classifier (0.802) showed no statistically significant difference with the AUC values obtained from the three radiologists (0.723, 0.733, and 0.734), with the p-values ranging from 0.066–0.109. When comparing the AUC value of the DCNN classifier with the pooled AUC value of the three readers (0.729), there was also no significant difference (*P* = 0.079) (Table 4, Fig. 1).

**Table 4:** Comparison of the diagnostic performances of the deep convolutional neural network classifier and three radiologists expressed as the area under the receiver operating characteristics curves using bootstrapping (1000 bootstrap samples).

	AUC	95% CI	P value^†^
DCNN	0.802	(0.733–0.872)	
Reader 1	0.733	(0.658–0.808)	0.109
Reader 2	0.723	(0.647–0.799)	0.066
Reader 3	0.734	(0.658–0.811)	0.122
Pooling^‡^	0.729	(0.657–0.796)	0.079

Abbreviations: AUC, area under the receiver operating characteristics curve; CI, confidence interval; DCNN, Deep convolutional neural network classifier.

^†^Comparison with DCNN.

^‡^Pooled performance of three readers calculated by multi-reader multi-case receiver operating characteristic analysis under the assumption of random readers and random cases.

Substantial agreement was seen among five-point confidence scale scores among the three radiologists (linear-weighted kappa statistics: 0.673, 0.664, and 0.740; Table 5).

**Table 5:** Inter-observer agreement on five-point confidence scale scores among the three radiologists

	Kappa value^†^	95% CI
Readers 1 and 2	0.6728	0.6090–0.7366 (substantial agreement)
Readers 1 and 3	0.6644	0.6027–0.7261 (substantial agreement)
Readers 2 and 3	0.7399	0.6923–0.7876 (substantial agreement)

Abbreviations: CI, confidence interval.

^†^We used the linear weighted kappa to account for partial agreement because the outcome encompasses ordinal scoring.

## Discussion

We demonstrated that our DL scoring classifier method based on the DCNN differentiated between tuberculous and pyogenic spondylitis to a degree comparable to the performance of three skilled radiologists. It was encouraging that the DCNN classifier had a similar AUC to that of the radiologists, considering the similarity between the MR findings of the two diseases^9^. Deep learning is known to outperform other feature-based machine learning algorithms in the medical imaging field^16,18–21^, as well as computer vision field. Consistent with previous studies, DCNN showed high performance in differentiating spondylitis. To the best of our knowledge, the present study may be the first to propose a deep-learning method for the differential diagnosis of infectious spondylitis using MR imaging.

Both tuberculous and pyogenic spondylitis require long-term use of appropriate antibiotics for proper treatment. Many cases of inappropriate treatment because of delayed diagnosis or misdiagnosis have been reported. Since it takes a long time for the treatment effect to appear, delayed diagnosis sometimes leads to an increased duration of hospitalization or increased need for surgery due to neurological deficits or spinal deformity^2,22^. Moreover, the relatively lower incidence of tuberculous spondylitis could increase the risk of misdiagnosis by less-experienced clinicians and radiologists. Tuberculosis is more common in underdeveloped countries^23^, in which the number of skilled radiologists is expected to be low. Therefore, the proposed DCNN may be helpful for low-experienced clinicians in underdeveloped countries. Additionally, we believe that our detection-based DCNN classifier could identify the lesions of tuberculous or pyogenic spondylitis on spine MR imaging taken without suspicion of infectious spondylitis.

We proposed a new method, DL score, for patient-based diagnosis rather than image-based diagnosis or lesion-based diagnosis. In previous studies about medical image analysis using deep learning, most studies were designed to reach a conclusion per image. However, in the actual reading process of MR images, a radiologist makes a decision for a patient after reviewing all lesions and slices of the MR imaging. In our study, training and validation were performed based on each lesion using object-detection DCNN. However, the final decision for each patient was made using DL score, which is a comprehensive quantitative value of all lesions detected within all slices of a MR imaging study. In our analysis, patient-based decisions using DL scoring showed significantly higher accuracy than slice-based decision. Accordingly, we monitored the saturation of the image-based accuracy together with the DL score accuracy to determine the learning saturation point of the model (Supplementary Fig. S1).

We have used various methods to mitigate the well-known problem of material shortage in deep learning of medical images. First, we used a pretrained DCNN model using a large dataset with fine-tuning for transfer learning, which has been reported to heighten diagnostic performance and generalizability than a model trained from scratch in radiologic decisions^13,24^. Transfer learning with fine tuning denotes restricted retraining of DCNN after pretraining on other large-scale datasets. This has been reported to be effective across heterogeneous datasets (between the ImageNet dataset and medical images), as well as homogeneous datasets^13^. Further, since transfer learning uses already learned volumes from a large amount of data, relatively good performance and high generalization has been reported, even with a small amount of target training material^13^. Second, image augmentation was used to reduce overfitting on the training dataset and to achieve generalizability^25^. Image augmentation is a way of enlarging training datasets using artificially created training images through various methods of image processing, such as random shadowing, flip, rotation, or shift. This technique has a great effect in deep learning study for relatively low-incidence diseases where large-scale imaging materials cannot be obtained^26^. In our study, we increased the training image set to 178 times through image augmentation (Supplementary Discussion 2). Finally, an additional benefit of the object detection model is an increase in learning material, because DCNN performs classification tasks as many as the number of lesions detected, not the total number of images^13,27^.

When developing a DL-powered, computer-aided, detection system, the presence of false positive findings is one of the most challenging issues. When reading screening mammography, false positive findings can have adverse effects, such as increased recall rate or prolongation of reading time^28^. In our pilot study, the inclusion of all small lesions in training resulted in an excessive increase in false positive findings. If our study was aimed at increasing detection sensitivity, such as lung nodule detection task, neglecting lesions below a certain size could have led to a great reduction in sensitivity. However, our task was to focus on the differentiation of two diseases, and it was important for the computer vision to be able to focus on the findings that are important for distinguishing the two diseases. Therefore, lesions smaller than a 1.5-cm^2^ bounding box were designed not to generate a ground-truth box, leading to an improvement in overall diagnostic performance.

There were several limitations to this study. First, the numbers of patients and MR images included were small. To compensate for this problem, we used image augmentation, transfer learning, and lesion-based learning. However, a multicenter study or validation using open datasets in public domains need to be conducted for a larger dataset.

Second, for spinal spondylitis, all examinations are recommended for clinical diagnosis, including microscopic or bacteriological examination, culture of the infected tissue, changes in clinical manifestations, radiological findings, blood and tissue cultures, and histopathological findings. However, in this study, only the diagnostic performance of MR imaging was evaluated. Although all clinical tests, including imaging, should be used for the final diagnosis, MR images can play an important role in determining the direction of the next procedure, before an invasive procedure, in the course of diagnosis^29^. Therefore, this study was conducted focusing on imaging diagnosis. In the future, it would be meaningful to make a comprehensive diagnosis by devising a DCNN that reflects clinical information together with image diagnosis.

Third, the MR imaging machines and pulse sequence parameters included in this study were not identical, and the quality of the MR images was heterogeneous. However, it was reported that DCNN can recognize images under various conditions and that the DCNN learned with images of various conditions recognizes unseen images more robustly^24^. We thought that various MR machines and protocols may help our DCNN classifier achieve a generalized vision.

Fourth, unlike standard practice in the radiologic reading environment, sagittal plane images could not be used in this study. Axial and sagittal plane images play different roles in complementing each other in differentiating between tuberculous and pyogenic spondylitis^3^. However, we only used axial-plane images due to the insufficient number of patients and various technical limitations. We suspect that images from various planes may improve the performance of the present study^30^.

Fifth, we did not assess the added value of the performance of the DCNN when incorporated with the radiologists. Since the performance of DCNN on the classification of spondylitis has not been previously shown, performance evaluation of DCNN itself was necessary before cooperating with radiologists.

Sixth, the retrospective design of our study inherently precluded us from demonstrating the effect of a new diagnostic tool on treatment outcomes. Although providing interpretation result of post-treatment MR imaging might be the indirect visualization of the patients’ outcome, post-treatment MR imaging is not performed in the majority of patients in our institution in accordance with the results of a previous investigation that showed post-treatment MR imaging does not correspond well with treatment outcomes and, thus, is not beneficial in the assessment of treatment outcomes in pyogenic spondylitis^31^. Although we could not evaluate clinical outcomes, we believe that our study demonstrates the potential added benefit for differential diagnosis of spondylitis using DCNN. Several studies have reported that a good prognosis can be obtained when appropriate antibiotic treatment is performed with early differential diagnosis of infectious spondylitis^2,32^.

In conclusion, we showed through this preliminary study that the DCNN classifier has comparable performance to that of skilled radiologists in differentiating between tuberculous and pyogenic spondylitis using MR images. A larger-scale study with further collection of multi-plane MR images needs to be performed for further validation of the DL scoring method proposed in this study.

## Methods

The institutional review board of Gangnam Severance Hospital approved this study (Approval No. 3-2017-0210). The MR images used for analysis were obtained during standard patient care, and thus, the institutional review board waived the need for informed consent from each patient due to our study’s retrospective design. All research was performed in accordance with relevant guidelines/regulations.

### Study Subjects

**Figure 2:**
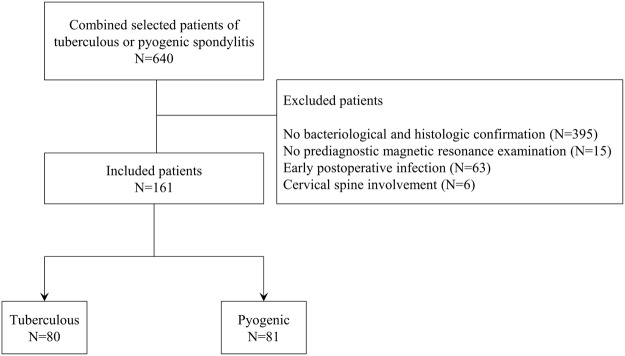
A flowchart of the patient selection process.

The subjects of this study comprised patients diagnosed with tuberculous or pyogenic spondylitis at Gangnam Severance Hospital from January 2007 to December 2016. We excluded patients whose diagnosis was not confirmed bacteriologically and/or histologically and those who had not undergone pre-diagnostic MR examination. Early postoperative infection cases were excluded: as the incidence of tuberculous spondylitis is extremely low in the postoperative period, it is unnecessary to differentiate between the two diseases. Patients who had cervical infectious spondylitis were excluded because the number of cases was small (tuberculous, 1 case; pyogenic, 5 cases) (Fig. 2). Details on confirmation tests and causative organism are provided in Supplementary Tables S2 and S3.

Finally, 80 patients with tuberculous spondylitis (151 MR examinations) and 81 patients with pyogenic spondylitis (143 MR examinations) were included in this study. MR examinations were performed with 1.5 T or 3 T MR machines. We chose the T2-weighted axial-plane image as the representative image for DCNN training. This is because more axial than sagittal images depicted lesions and because extraspinal lesions were well contained, which is important for DCNN training. Supplementary Table S4 summarizes the rough parameters for the MR pulse sequence.

### Image Preprocessing

**Figure 3:**
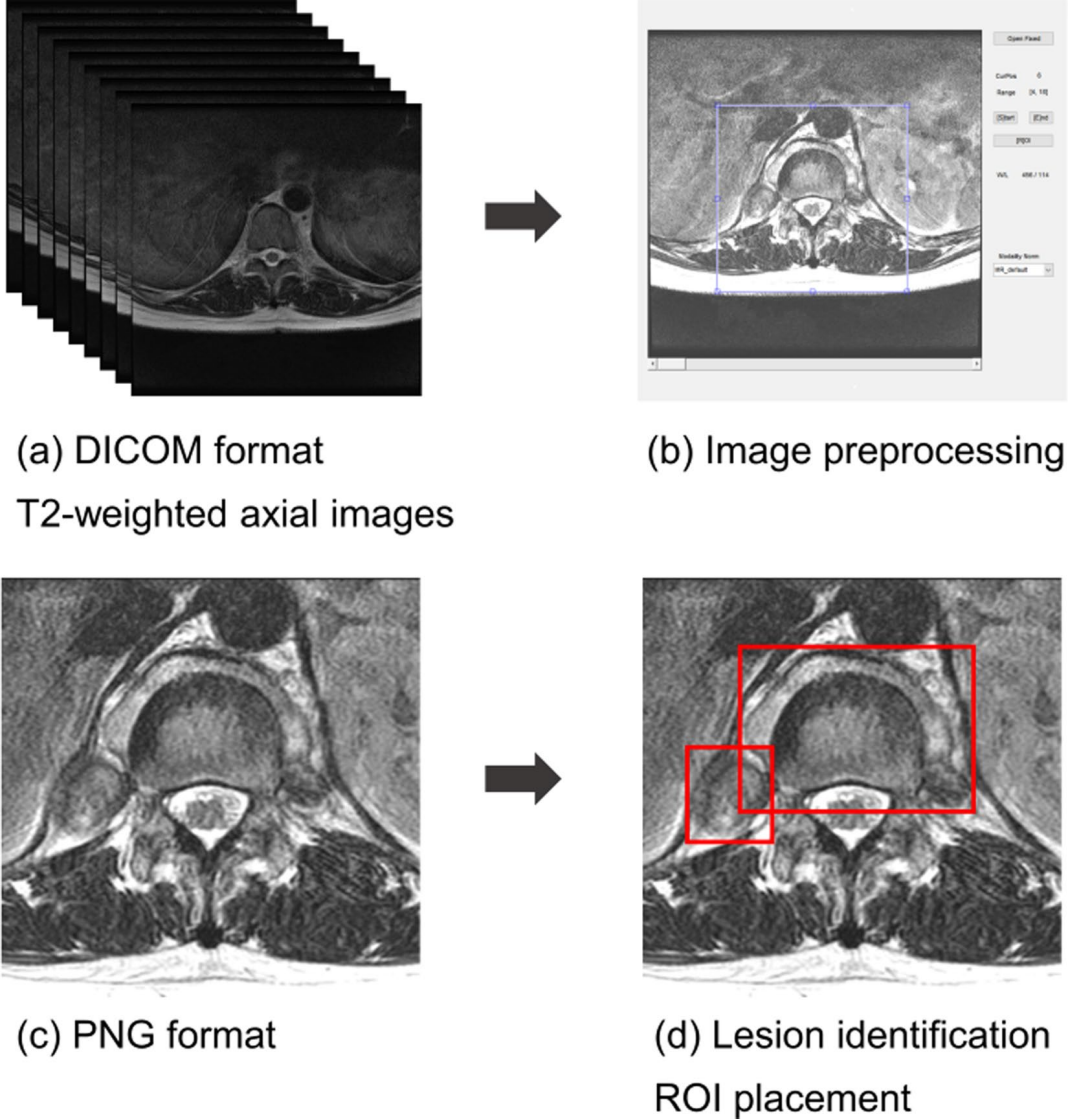
A flowchart of the study process from image preprocessing to ground-truth making. The image of step (**c**) and the ground-truth box of step (**d**) were used as input data for the training phase. Abbreviations: DICOM, Digital Imaging and Communications in Medicine; PNG, Portable Network Graphics; ROI, Region of Interest.

We developed in-house software for image preprocessing based on MATLAB (R2017a Version 9.2; Mathworks, Natick, MA). A radiologist performed image preprocessing through the software (Fig. 3) by selecting a 10 × 10 square centimeter on the lesion-containing axial image, such that the vertebral body was at the center of the square, to generate an image for DCNN training. The generated images were saved in PNG format (300 × 300 pixel size, 24 bits color).

### Lesion Masking and Ground-Truth Box Generation

Two radiologists identified lesions by consensus and manually drew minimum rectangle regions of interests (ROIs) enclosing each lesion, which was called a bounding box and was used as the ground truth. Lesions smaller than 1.5 cm^2^ were ignored without drawing a ground-truth box because the results of our pilot study showed that lesions less than 1.5 cm^2^ in size only caused false positive detection and decreased overall diagnostic performance. Images that did not contain a ground-truth box were excluded from the study. The total number of images obtained through these procedures was 1901 for tuberculous spondylitis and 1588 for pyogenic spondylitis. Figure 3 summarizes the flow from image processing to lesion tagging. The images and ground-truth boxes created through these steps were used for DCNN training. ROI placement was performed using MIPAV (Medical Image Processing, Analysis and Visualization, version 7.4.0; National Institutes of Health, Bethesda, MD).

### DCNN Architecture Construction

The Python programming language 3.6.1 (https://www.python.org) and the Tensorflow 1.3.0 framework (https://www.tensorflow.org) were used to construct the DCNN architecture. We used the Single Shot Multibox Detector 300 model for the DCNN architecture, object detection, and classification model to perform lesion-by-lesion diagnosis. Fine-tuning on DCNN was performed based on the pretrained volume, which was pretrained on the Pattern Analysis, Statistical Modelling and Computational Learning Visual Object Classes (PASCAL VOC 2007) dataset^33^. Training and validation of DCNN was performed using a computer with a GeForce GTX 1080 Ti (NVIDIA, Santa Clara, CA) graphics processing unit, a Core i7-7700K 4.2 GHz (Intel, Santa Clara, CA) central processing unit, and 16 GB of random access memory. Details on the DCNN architecture construction are provided in Supplementary Discussion 1.

### Generalization of DCNN in Spondylitis Classification - Training and Validation

We evaluated the classification performance of our DCNN classifier with four-fold cross validation. Two (tuberculous and pyogenic) groups were randomly partitioned into four equal-sized independent subsets patient-wise instead of image-wise. First, the preprocessed images and corresponding ground-truth boxes of the three subsets were used for DCNN training. In the validation phase, the diagnostic performance of the binary classification (tuberculous or pyogenic) was measured using the remaining independent subset. The same cross validation process was repeated four times.

### Deep-Learning (DL) Score

**Figure 4:**
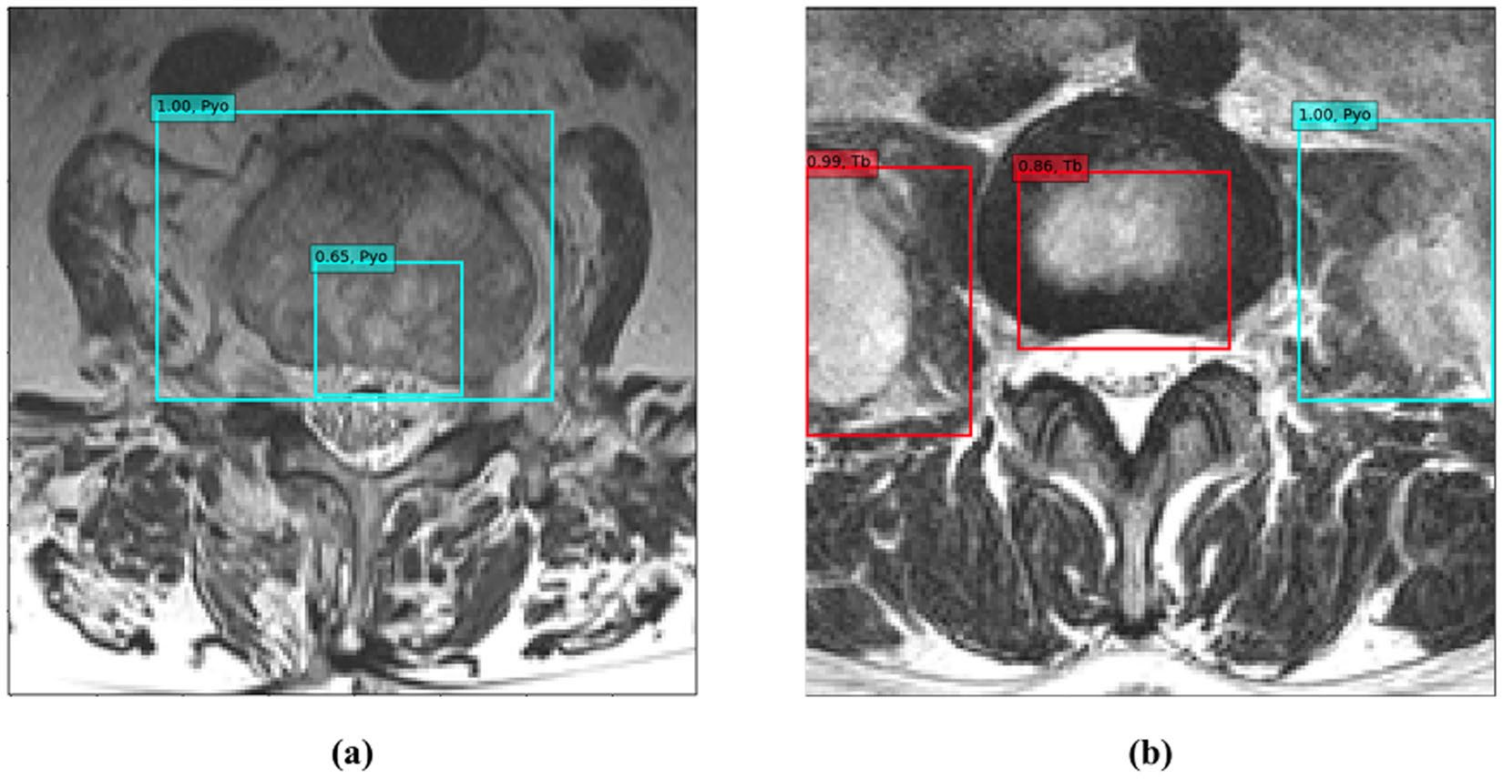
Two examples of probability measurements within each image. (**a**) A 72-year-old woman with pyogenic spondylitis. Sky-blue boxes indicate pyogenic lesions detected by the deep convolutional neural network classifier. The probability of pyogenic spondylitis for this image is 1.00, which is the highest value among the detected pyogenic lesions. Because no tuberculous lesion is observed, the probability of tuberculous spondylitis is 0. (**b**) A 31-year-old woman with tuberculous spondylitis. Two red boxes indicate tuberculous lesions and a sky-blue box indicates a pyogenic lesion. The probability of tuberculous and pyogenic spondylitis is 0.99 and 1.00, respectively. Abbreviations: Pyo, pyogenic spondylitis; Tb, tuberculous spondylitis.

**Figure 5:**
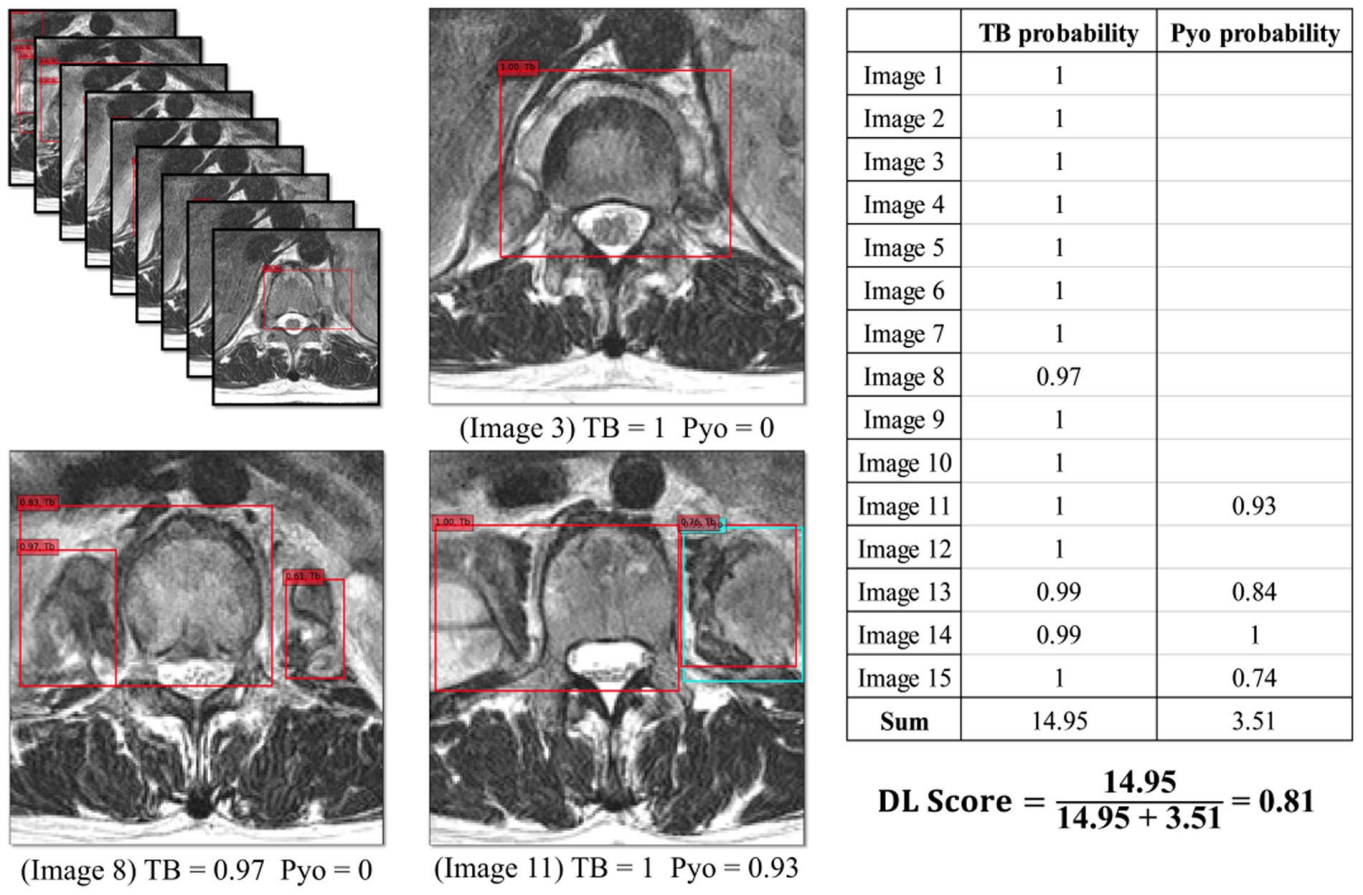
An example of deep-learning scoring in a 30-year-old woman with tuberculous spondylitis patients. The trained deep convolutional neural network classifier detected lesions in each image and displayed their position using a rectangular box. Each box was divided into two colors according to the class of the lesion, and a probability was given (tuberculous spondylitis, red box; pyogenic spondylitis, sky-blue box). The highest probabilities for tuberculous and pyogenic spondylitis obtained from each magnetic resonance image were summed over all slices, respectively. The deep-learning score was defined as the proportion of the summed probability for tuberculosis to the summed probability for tuberculosis and pyogenic spondylitis. The final deep-learning score was calculated as 0.81, which was higher than the selected threshold value (0.31, Youden index), and was diagnosed as tuberculous spondylitis. Abbreviations: DL, deep-learning; Pyo, pyogenic spondylitis; Tb, tuberculous spondylitis.

Our classifier was designed to calculate DL scores for each patient to make a comprehensive decision on the patient based on the decisions of the lesions the patient had. The highest probabilities for tuberculous and pyogenic spondylitis were measured in each slice, respectively (Fig. 4). Then, the probabilities for those two diseases were summed over all slices. The final decision was determined by the DL score (the proportion of the summed probability for tuberculous to the summed probability for tuberculous and pyogenic spondylitis) using the following equation: DL score = summed tuberculous score/(summed tuberculous score + summed pyogenic score); summed tuberculous score = sum of the selected tuberculous probability over all slices; and summed pyogenic score = sum of the selected pyogenic probability over all slices. Figure 5 summarizes the process of DL scoring from the MR images of a patient with tuberculous spondylitis.

### Image Analysis by the Radiologists

Images were independently evaluated by three board-certificated musculoskeletal radiologists (10, 9, and 7 years of experience in musculoskeletal imaging interpretation, respectively). A preprocessed T2-weighted axial image stack for each patient was provided, which was identical to that used during DCNN training. The three radiologists were instructed to provide a five-point confidence scale score for the question “Is this patient’s disease tuberculous spondylitis rather than pyogenic spondylitis?” based on the MR imaging.

### Statistical and Data Analysis

Independent-samples *t* test was used to compare continuous variables that were normally distributed. The Pearson chi-square test was used to compare categorical variables. Fisher’s exact test was applied for categorical variables when the expected frequencies for any group were less than or equal to five.

The results of the validation set and the average results of the four-fold cross validation of the DCNN classifier were measured based on the patient, not on the image. The Delong method was used to calculate the AUCs for the DCNN classifier and three radiologists, and multi-reader multi-case ROC analysis was used to calculate the pooled AUC of three radiologists under the assumption of random readers and random cases^34,35^. Bootstrapping (1000 bootstrap samples) was used to compare the AUCs of the DCNN classifier and three radiologists^36^. Sensitivity, specificity and accuracy were determined from the optimal threshold using the Youden index (Youden index = sensitivity + specificity − 1).

Kappa statistics were used to analyze agreement among the three radiologists. We used the linear weighted kappa to account for partial agreement because the outcome encompasses ordinal scoring. Interpretation of the kappa values was based on a guidelines provided by Landis and Koch^37^.

All *P* values less than 0.05 were considered to indicate statistical significance. All statistical analyses were conducted using IBM SPSS Statistics (version 23.0; IBM Corporation, Armonk, NY) and R (version 3.4.1; R Foundation for Statistical Computing, Vienna, Austria).

## Electronic supplementary material

Supplementary information

## Data Availability

The datasets generated during and/or analyzed during the current study are available from the corresponding author on reasonable request.

## References

[1] Cottle L, Riordan T (2008). Infectious spondylodiscitis. Journal of Infection.

[2] Garg RK (2013). Evaluation of prognostic factors in medically treated patients of spinal tuberculosis. Rheumatol Int.

[3] Jung NY, Jee WH, Ha KY, Park CK, Byun JY (2004). Discrimination of tuberculous spondylitis from pyogenic spondylitis on MRI. AJR Am J Roentgenol.

[4] Harada Y, Tokuda O, Matsunaga N (2008). Magnetic resonance imaging characteristics of tuberculous spondylitis vs. pyogenic spondylitis. Clin Imaging.

[5] Chang MC, Wu HT, Lee CH, Liu CL, Chen TH (2006). Tuberculous spondylitis and pyogenic spondylitis: comparative magnetic resonance imaging features. Spine (Phila Pa 1976).

[6] Galhotra RD, Jain T, Sandhu P, Galhotra V (2015). Utility of magnetic resonance imaging in the differential diagnosis of tubercular and pyogenic spondylodiscitis. J Nat Sci Biol Med.

[7] Yeom JA, Lee IS, Suh HB, Song YS, Song JW (2016). Magnetic Resonance Imaging Findings of Early Spondylodiscitis: Interpretive Challenges and Atypical Findings. Korean J Radiol.

[8] Yoon YK (2015). Differential diagnosis between tuberculous spondylodiscitis and pyogenic spontaneous spondylodiscitis: a multicenter descriptive and comparative study. The Spine Journal.

[9] Kim CJ (2010). A comparative study of pyogenic and tuberculous spondylodiscitis. Spine (Phila Pa 1976).

[10] Krizhevsky, A., Sutskever, I. & Hinton, G. E. Imagenet classification with deep convolutional neural networks. In *Advances in neural information processing systems* 1097–1105 (2012).

[11] LeCun Y, Bottou L, Bengio Y, Haffner P (1998). Gradient-based learning applied to document recognition. Proceedings of the IEEE.

[12] Russakovsky O (2015). ImageNet Large Scale Visual Recognition Challenge. International Journal of Computer Vision.

[13] Lakhani, P. & Sundaram, B. Deep Learning at Chest Radiography: Automated Classification of Pulmonary Tuberculosis by Using Convolutional Neural Networks. *Radiology*, 162326 (2017). 10.1148/radiol.2017162326 28436741

[14] Roth, H. R. *et al*. A new 2.5 D representation for lymph node detection using random sets of deep convolutional neural network observations. In *International Conference on Medical Image Computing and Computer-Assisted Intervention* 520–527 (Springer, 2014). 10.1007/978-3-319-10404-1_65 PMC4295635 25333158

[15] Trebeschi, S. *et al*. Deep Learning for Fully-Automated Localization and Segmentation of Rectal Cancer on Multiparametric MR. *Scientific Reports* **7** (2017). 10.1038/s41598-017-05728-9 PMC5509680 28706185

[16] Ghafoorian, M. *et al*. Location sensitive deep convolutional neural networks for segmentation of white matter hyperintensities. *Sci Rep* **7**, 10.1038/s41598-017-05300-5 (2017). 10.1038/s41598-017-05300-5 PMC5505987 28698556

[17] Lee JG (2017). Deep Learning in Medical Imaging: General Overview. Korean J Radiol.

[18] Wang J (2016). Discrimination of Breast Cancer with Microcalcifications on Mammography by Deep Learning. Sci Rep.

[19] Cheng JZ (2016). Computer-Aided Diagnosis with Deep Learning Architecture: Applications to Breast Lesions in US Images and Pulmonary Nodules in CT Scans. Sci Rep.

[20] Choi H, Jin KH (2018). Predicting cognitive decline with deep learning of brain metabolism and amyloid imaging. Behav Brain Res.

[21] Rachmadi MF, Valdés-Hernández MdC, Agan MLF, Komura T (2017). Deep Learning vs. Conventional Machine Learning: Pilot Study of WMH Segmentation in Brain MRI with Absence or Mild Vascular Pathology. Journal of Imaging.

[22] McHenry MC, Easley KA, Locker GA (2002). Vertebral Osteomyelitis: Long-Term Outcome for 253 Patients from 7 Cleveland-Area Hospitals. Clinical Infectious Diseases.

[23] Organization, W. H. Global tuberculosis report 2016. (2016).

[24] Shin H-C (2016). Deep convolutional neural networks for computer-aided detection: CNN architectures, dataset characteristics and transfer learning. IEEE transactions on medical imaging.

[25] Wong, S. C., Gatt, A., Stamatescu, V. & McDonnell, M. D. Understanding data augmentation for classification: when to warp? In *Digital Image Computing: Techniques and Applications (DICTA), 2016 International Conference on* 1–6 (IEEE, 2016).

[26] Garg RK, Somvanshi DS (2011). Spinal tuberculosis: a review. J Spinal Cord Med.

[27] Larson DB (2018). Performance of a Deep-Learning Neural Network Model in Assessing Skeletal Maturity on Pediatric Hand Radiographs. Radiology.

[28] Kim SJ, Moon WK, Seong MH, Cho N, Chang JM (2009). Computer-aided detection in digital mammography: false-positive marks and their reproducibility in negative mammograms. Acta Radiol.

[29] Sharif HS (1992). Role of MR imaging in the management of spinal infections. AJR Am J Roentgenol.

[30] Ju, C., Bibaut, A. & van der Laan, M. The relative performance of ensemble methods with deep convolutional neural networks for image classification. *Journal of Applied Statistics*, 1–19, 10.1080/02664763.2018.1441383 (2018). 10.1080/02664763.2018.1441383 PMC6800663 31631918

[31] Kowalski TJ (2007). Follow-up MR imaging in patients with pyogenic spine infections: lack of correlation with clinical features. AJNR Am J Neuroradiol.

[32] Wu W (2017). Improvement in clinical outcome and infection control using molecular diagnostic techniques for early detection of MDR tuberculous spondylitis: a multicenter retrospective study. Emerg Microbes Infect.

[33] Liu, W. *et al*. Ssd: Single shot multibox detector. In *European conference on computer vision* 21–37 (Springer, 2016).

[34] DeLong ER, DeLong DM, Clarke-Pearson DL (1988). Comparing the areas under two or more correlated receiver operating characteristic curves: a nonparametric approach. Biometrics.

[35] Obuchowski NA (2004). Multireader, multicase receiver operating characteristic analysis: an empirical comparison of five methods. Acad Radiol.

[36] Rutter CM (2000). Bootstrap estimation of diagnostic accuracy with patient-clustered data. Academic Radiology.

[37] Landis, J. R. & Koch, G. G. The measurement of observer agreement for categorical data. *Biometrics*, 159–174 (1977). 843571

