# Supplementary material for: Performance of the deep convolutional neural network based magnetic resonance image scoring algorithm for differentiating between tuberculous and pyogenic spondylitis
Source: Sci Rep. 2018 Sep 3;8:13124. doi: 10.1038/s41598-018-31486-3 (PMC6120953; doi:10.1038/s41598-018-31486-3)
Supplement: Supplementary file 1 — Supplementary information [file 41598_2018_31486_MOESM1_ESM.docx]

**Performance of the deep convolutional neural network based magnetic resonance image scoring algorithm for differentiating between tuberculous and pyogenic spondylitis**

Kiwook Kim, MD^1+^, Sungwon Kim, MD^2+^, Young Han Lee, MD, PhD^2^, Seung Hyun Lee, MD^3^, Hye Sun Lee, PhD^4^, and Sungjun Kim, MD, PhD^1*^

^1^Department of Radiology, Gangnam Severance Hospital, Yonsei University College of Medicine, Research Institute of Radiological Science, Center for Clinical Imaging Data Science

^2^Department of Radiology, Severance Hospital, Yonsei University College of Medicine, Research Institute of Radiological Science, Center for Clinical Imaging Data Science

^3^Department of Radiology, National Health Insurance Service Ilsan Hospital

^4^Biostatistics Collaboration Unit, Research Center for Future Medicine, Yonsei University College of Medicine

^+^Kiwook Kim and Sungwon Kim contributed equally to this work as co-first authors.

*Correspondence and requests for materials should be addressed to Sungjun Kim (email: [AGN70@yuhs.ac](mailto:AGN70@yuhs.ac))

**Supplementary Discussion 1.**

**Deep learning with Single Shot Multibox Detector**

The field of deep convolutional neural networks (DCNNs) for image recognition is divided into several major categories, and accordingly, there are various competitions in the field of computer vision (ILSVRC, PASCAL VOC Challenge, LifeCLEF Bird Identification Task, COCO Image Segmentation Challenge, and so on)^1-4^.

- Classification: the task of classifying what appears in an image into one out of a set of pre-defined classes
- Object detection & classification: the task of localizing where objects are in an image and classifying
- Segmentation: the task of classifying each pixel in an image into one out of a set of pre-defined classes and transforming the pixels of the image into a set of outlines or polygons

AlexNet, Inception, and ResNet, which are mentioned in many other articles^5,6^, are known as models for classification. Single Shot Multibox Detector (SSD), Yolo, and RetinaNet are known as state-of-the-art models in object detection and classification.

In this paper, we used SSD, which is a multi-layer feed-forward DCNN and of use for object detection and classification. SSD has a unique data input/output structure. The input is a single image similar to other models, but the output shows information about the location, size, and type of objects detected within a given image at a time. The difference in the classification model is that it is possible to obtain not only the kind of object but also positional information and to find many objects at the same time. For example, in the case of using SSD in this study, multiple lesions of each image were detected at the same time; the positions of the lesions were displayed; and the lesions were classified as tuberculous or pyogenic spondylitis, respectively. The advantages of the SSD feature mentioned above are as follows: (1) Using the SSD model, we can find the location of the object in the image with classification. In other words, it is possible to see which location of the image DCNN used as the basis of judgement, by presenting the location of the lesion as an output. (2) It is possible to increase the number of training data for SSD by detecting multiple lesions present in each image.

Another advantage for using this model is that the diagnostic performance could be improved through the following process: the classes and locations of all lesions are detected in the magnetic resonance (MR) images of a patient, and all the obtained information of multiple lesions is integrated to make a final diagnosis of the patient. This approach was thought to be closer to the diagnostic approach of radiologists in the actual medical field, which involves the process of detecting lesions first in all images and subsequently synthesizing the information to make a final diagnosis. Since SSD only provides results for each image, not for a patient, this paper proposes a method called deep-learning (DL) score to compensate for this weakness. A DL score of a patient was calculated from the confidence score of lesions in all images detected by SSD. Based on a specific cut-off of DL score, patients were diagnosed with tuberculous or pyogenic spondylitis. The role of DL score is similar to the process by which a radiologist synthesizes the information gained from all images to make a final diagnosis.

Supplementary Figure S5 shows the architecture of the SSD. In this figure, the part marked “Conv” is a convolution block and contains a number of convolutional layers inside. This SSD-based network belongs to the “deep” convolutional neural network because they have multiple hidden convolutional layers that are layers in between input and output layers. Our SSD is configured using a portion of the Oxford group's VGG-16 classification model^7^, but there are some important differences. First, we derive the prediction results at various points in the middle of the network, so we use all of the information of various scales (multiscale) from the detailed to the large parts of an input image. This makes it possible to detect the same object repeatedly on various scales. To solve this problem, non-max suppression is performed at the last step, and the object with the highest class probability is selected as the answer. Second, we used transfer learning to compensate for the relatively small number of images used in the training set. The transfer learning refers to the use of different large-volume data for pre-learning. When performing additional learning using the target data, it showed good performance, compared with no prior learning^5,8^. In this study, SSD was pretrained using published data from the VOC 2007 dataset^2^ (total 9,963 images, containing 24,640 annotated objects) and then learned using MR images of spondylitis patients. At this time, layers from Conv1 to Conv4 were fixed to the value at the time of pre-training completion, and the subsequent convolutional layers were learned in the training phase. This process is referred to as fine-tuning^9^.

**Supplementary Discussion 2.**

**Imaging augmentation**

The most common technique to reduce overfitting on training data is to artificially enlarge the training dataset using additional synthetically modified data, called “data augmentation.” The following image augmentation technique was applied in our study: random brightness alteration in the range of ±20%, random contrast alteration in the range of ±20%, random lighting in the range of ±20%, and horizontal flip with a probability of 0.5. Arithmetically, image augmentation can increase the number of training image sets up to 128,000 times (40 x 40 x 40 x 2). In our study, an artificial image was created for each image in each epoch, and a new image set corresponding to the number of actual training image sets was created for each epoch. When the final 178 epochs were completed, the training image set was increased by 178 times.

**Supplementary Figure S1.** Changes in image and patient-specific accuracy as the epoch changes during deep learning training (deep-learning score cutoff = 0.5).

acc(t) = image-specific accuracy in training set; acc(p) = patient-specific accuracy in validation set; acc(v) = image-specific accuracy in validation set


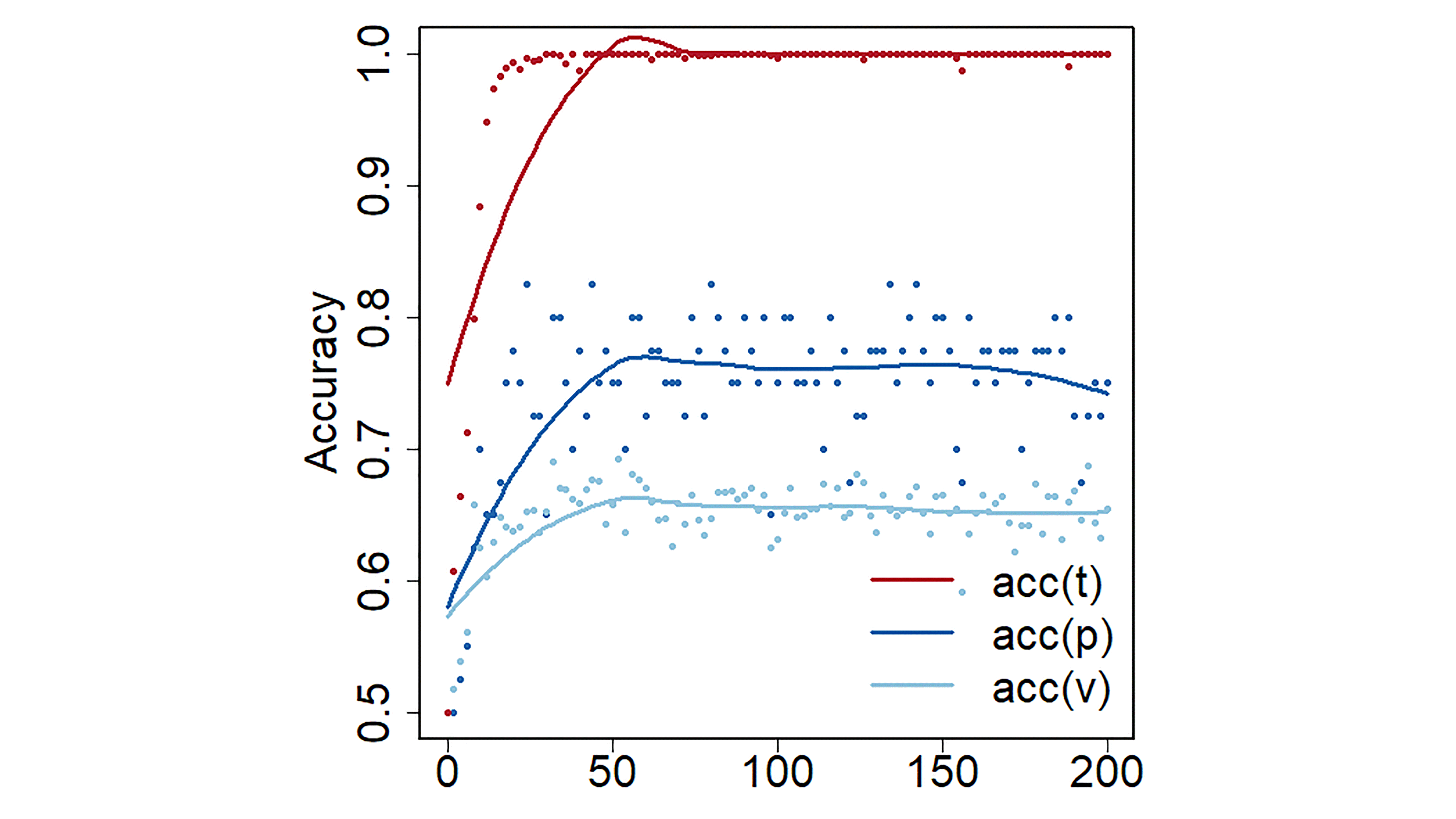


**Supplementary Table S2.** Confirmation tests in 161 patients with tuberculous and pyogenic spondylitis

| **Confirmation test** | N (%) |
| --- | --- |
| **Tuberculous spondylitis** | 80 |
| Surgery | 56 (70.0) |
| Histological examination | 23 (28.8) |
| Bacteriological examination | 30 (37.5) |
| TB-PCR test | 3 (3.8) |
| Percutaneous bone biopsy | 21 (26.3) |
| Histological examination | 6 (7.5) |
| Bacteriological examination | 15 (18.8) |
| Abscess aspiration^†^ | 3 (3.8) |
| **Pyogenic spondylitis**^†^ | 81 |
| Surgery | 55 (67.9) |
| Percutaneous bone biopsy | 10 (12.3) |
| Blood culture | 14 (17.3) |
| Abscess aspiration | 2 (2.5) |

TB-PCR, mycobacterium tuberculosis-polymerase chain reaction

† These groups were confirmed only by bacteriological examination.

**Supplementary Table S3.** Causative organisms identified in 81 patients with pyogenic spondylitis

| **Causative organism** | 81 (%) |
| --- | --- |
| **Gram-positive cocci** |  |
| Staphylococcus spp. | 41 (49.4) |
| Staphylococcus aureus | 33 (40.7) |
| Coagulase-negative staphylococci | 8 (9.9) |
| Streptococcus spp. | 13 (16.0) |
| Viridans streptococci | 4 (4.9) |
| Streptococcus pneumoniae | 1 (1.2) |
| Streptococcus agalactiae | 8 (9.9) |
| Other streptococcus spp. | 6 (7.4) |
| Enterococcus spp. | 2 (2.5) |
| **Gram-positive bacilli** |  |
| Diphtheroids | 1 (1.2) |
| **Gram-negative rod** |  |
| Escherichia coli | 9 (11.1) |
| Klebsiella pneumoniae | 2 (2.5) |
| Acinetobacter baumannii | 2 (2.5) |
| Nontyphoidal Salmonella | 1 (1.2) |
| Stenotrophomonas maltophilia | 1 (1.2) |
| Enterobacter aerogenes | 1 (1.2) |
| **Gram-negative bacilli** |  |
| Prevotella loescheii | 1 (1.2) |
| **Gram-negative diplococci** |  |
| Neisseria sp. | 1 (1.2) |

spp., species; sp., species

**Supplementary Table S4.** Summary of Magnetic Resonance Pulse Sequence Parameters

| Tesla (T) | 1.5 | 3 |
| --- | --- | --- |
| Acquisition type | 2D TSE | 2D FSE / 2D TSE |
| Weighting | T2 | T2 |
| Repetition time (msec) | 2810-4500 | 2932-4657 |
| Echo time (msec) | 118-120 | 103.5-120 |
| Frequency-encoding matrix | 384-448 | 320-340 |
| Phase-encoding matrix | 202-254 | 224-347 |
| Section thickness/gap (mm) | 3/0.3 or 4/0.4 | 3/0.15 or 4/0.4 |
| Number of signals acquired | 3 | 2 or 3 |

FSE: fast spin echo

TSE: turbo spin echo

**Supplementary Figure S5.** Conceptual architecture of the Single Shot Multibox Detector model used in this study.


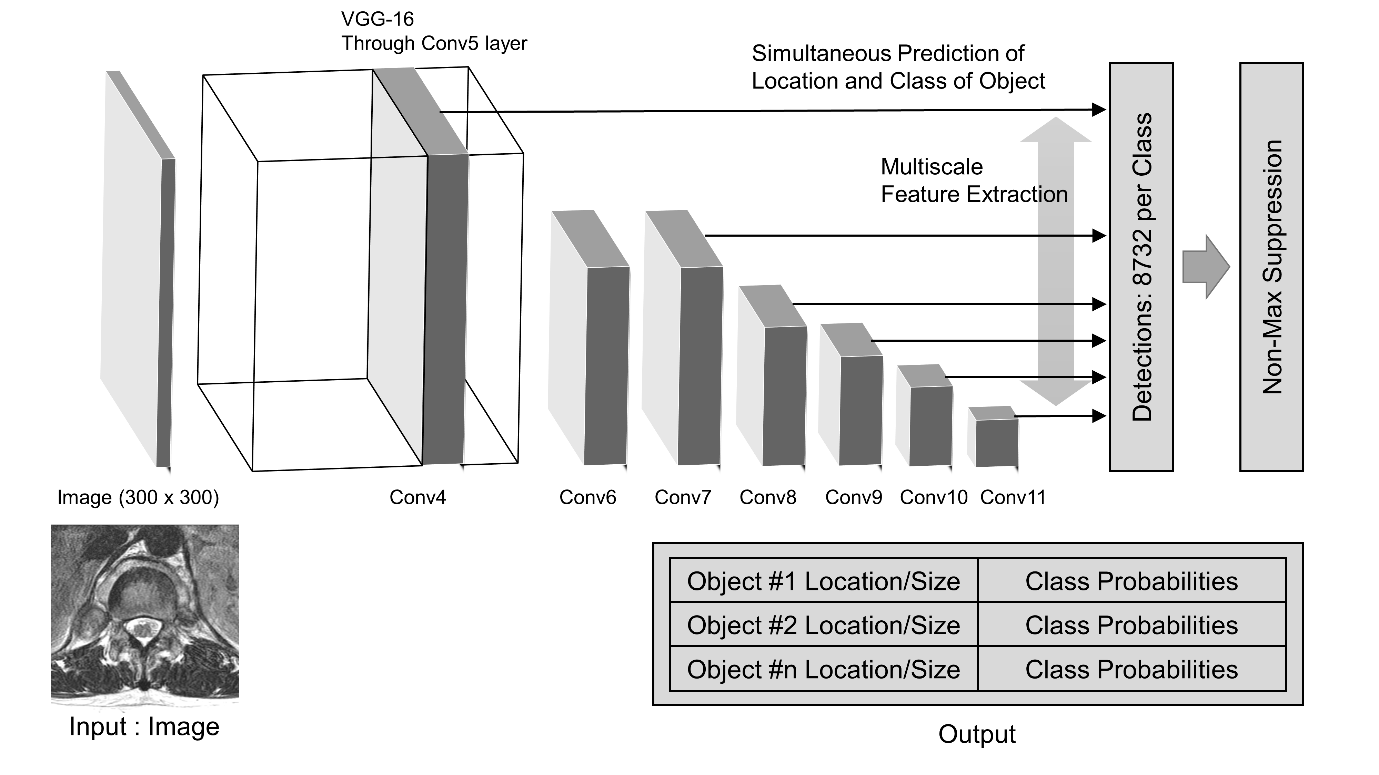


**Supplementary References**

1. Russakovsky, O. *et al.* ImageNet Large Scale Visual Recognition Challenge. *International Journal of Computer Vision* **115**, 211-252, <https://doi.org/10.1007/s11263-015-0816-y> (2015).

2. Everingham, M., Van Gool, L., Williams, C. K. I., Winn, J. & Zisserman, A. The Pascal Visual Object Classes (VOC) Challenge. *International Journal of Computer Vision* **88**, 303-338, <https://doi.org/10.1007/s11263-009-0275-4> (2010).

3. Goëau, H. *et al.* LifeCLEF Bird Identification Task 2014 in *CLEF: Conference and Labs of the Evaluation Forum* 2014).

4. Lin, T.-Y. *et al.* Microsoft COCO: Common Objects in Context 740-755 (Springer International Publishing, 2014).

5. Lakhani, P. and Sundaram, B. Deep Learning at Chest Radiography: Automated Classification of Pulmonary Tuberculosis by Using Convolutional Neural Networks. *Radiology* **284**, 574-582, <https://doi.org/10.1148/radiol.2017162326> (2017).

6. Kang, G., Liu, K., Hou, B. & Zhang, N. 3D multi-view convolutional neural networks for lung nodule classification. *PLoS One* **12**, e0188290, <https://doi.org/10.1371/journal.pone.0188290> (2017).

7. Simonyan, K. and Zisserman, A. Very deep convolutional networks for large-scale image recognition. *arXiv preprint arXiv:1409.1556* (2014).

8. Shin, H. C. *et al.* Deep Convolutional Neural Networks for Computer-Aided Detection: CNN Architectures, Dataset Characteristics and Transfer Learning. *IEEE Trans Med Imaging* **35**, 1285-1298, <https://doi.org/10.1109/tmi.2016.2528162> (2016).

9. Becherer, N., Pecarina, J., Nykl, S. & Hopkinson, K. Improving optimization of convolutional neural networks through parameter fine-tuning. *Neural Computing and Applications*, <https://doi.org/10.1007/s00521-017-3285-0> (2017).
